# Analysis of Influenza and RSV dynamics in the community using a ‘Local Transmission Zone’ approach

**DOI:** 10.1038/srep42012

**Published:** 2017-02-09

**Authors:** Gal Almogy, Lewi Stone, B. Andrei Bernevig, Dana G. Wolf, Marina Dorozko, Allon E. Moses, Ran Nir-Paz

**Affiliations:** 1 Flurensics Inc., Tel Aviv, 64101 Israel; 2 School of Mathematical and Geospatial Sciences, RMIT University, Melbourne, Australia; 3 Department of Zoology, Faculty of Life Sciences, Tel Aviv University, Tel Aviv, Israel; 4 Department of Physics, Princeton University, Princeton, NJ 08544, USA; 5 Department of Clinical Microbiology and Infectious Diseases, Hadassah-Hebrew University Medical Center, Jerusalem 91120, Isreal.

## Abstract

Understanding the dynamics of pathogen spread within urban areas is critical for the effective prevention and containment of communicable diseases. At these relatively small geographic scales, short-distance interactions and tightly knit sub-networks dominate the dynamics of pathogen transmission; yet, the effective boundaries of these micro-scale groups are generally not known and often ignored. Using clinical test results from hospital admitted patients we analyze the spatio-temporal distribution of Influenza Like Illness (ILI) in the city of Jerusalem over a period of three winter seasons. We demonstrate that this urban area is not a single, perfectly mixed ecology, but is in fact comprised of a set of more basic, relatively independent pathogen transmission units, which we term here Local Transmission Zones, LTZs. By identifying these LTZs, and using the dynamic pathogen-content information contained within them, we are able to differentiate between disease-causes at the individual patient level often with near-perfect predictive accuracy.

Seasonal epidemics of Influenza-Like Illness (ILI) are a major source of winter morbidity typically affecting some 10–50% of a given population^1^,^2^,^3^,^4^,^5^. While the Influenza virus may cause ILI, there are many other respiratory diseases such as Respiratory Syncytial Virus (RSV), ParaInfluenza, Adenovirus, and other pathogens that may be equally responsible^6^,^7^,^8^,^9^,^10^. Disease manifestations of ILI range across the spectrum from mild presentation to acute respiratory failure^11^. The spatio-temporal dynamics of the different pathogens that can lead to ILI, and their interactions, is an area of research for which little is known. Recent work has emphasized that transmission is tightly linked to the underlying structure of the host community: a complex, dynamic network of short- and long-distance social interactions. The long-distance interactions facilitate disease transmission over great geographic distances^12^,^13^, for example via international airplane routes, and can play an important role in controlling the global distribution and spread of ILI and pandemics^14^,^15^.

However, the fundamental processes of pathogen transmission are found at much smaller geographic scales, since close physical proximity between infected and susceptible individuals is a requirement for occurrence of disease^16^,^17^. This is a regime where short-distance interactions dominate, and tightly knit sub-networks or ‘cliques’^18^ such as families, kindergartens or schools are the high-risk groups in the population^19^,^20^. The effective boundaries of these micro-scale groups are generally not known, and it is difficult to determine which groups within a large community form a pathogen’s basic ‘epidemiological unit.’ Moreover little is known about the spatio-temporal dynamics of ILI at these smaller spatial scales. Here we develop new methods that focus on the unexplored patterns and dynamics of ILI epidemics on these smaller scales by developing a new theory of ‘Local Transmission Zones’ (LTZs). We demonstrate how a single urban area can be subdivided into a set of smaller geographic zones, that are relatively independent of one another and each individually may be considered as a pathogen’s basic epidemiological unit. The disease dynamics within the urban area cannot be fully understood without subdividing it into its subcomponent LTZs. Our study outlines a new approach for identifying LTZs and explores their properties with regard to pathogen transmission.

LTZs are defined by the property that the transmission rate of an infectious disease within an LTZ is significantly greater than the average transmission rate of the disease between the different LTZs. This means that the population within an LTZ is in relative terms highly connected, and we suppose sufficiently connected to justify the assumption of ‘random mixing,’ so that an invading pathogen could come into contact reasonably rapidly with most of members of the LTZ. However, an invading pathogen may have difficulty in spreading beyond the confines of the relatively isolated LTZ. Extreme examples of LTZs have been documented in the literature and include confined military bases or cruise ships^21^,^22^,^23^,^24^ or other small isolated communities (e.g. religious or ethnic) through which diseases rapidly propagate, reaching most members of the population.

To investigate the possibility of LTZs in a large regional area, we analyze clinical data from a healthcare medical center in the city of Jerusalem, containing the clinical test results for Influenza virus and Respiratory Syncytial Virus (RSV) from patients presenting with ILI symptoms. Using a ‘k-means clustering’ algorithm, putative LTZ groups are identified solely based on the physical distance between home locations of the patients. The methodology is akin to the procedure of ‘community detection’ applied in the study of complex networks and designed to locate highly connected clusters of nodes^25^,^26^. Our analysis finds that while Influenza and RSV incidences tend to overlap and show more or less equal number of cases over the whole region, individual LTZs show a far more homogeneous disease content at most given times, with some being dominated by RSV while others by Influenza. We use these findings to arrive at a prediction algorithm that, applied to patients presenting at the hospital with ILI, is capable of differentiating between cases of Influenza and RSV, often with near-perfect accuracy.

## Results

### Defining Local Transmission Zones

The transmission dynamics of respiratory pathogens in a population are constrained by the physical distance between infected and susceptible individuals. An LTZ for a given pathogen represents a group of individuals within the general population, such that the transmission probability of the pathogen within the group is greater than transmission probability between that group and any of the other groups. Thus we suppose the population of a region can be subdivided into a set of *k* groups or LTZs, such that for any two LTZs *i* and *j*:


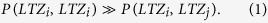


Here *P*(*LTZ*_*i*_, *LTZ*_*j*_) is the average probability that an individual from *LTZ*_*i*_ infects an individual from *LTZ*_*j*_.

In our case we are given the geographic coordinates of a group of individuals, and we assume that the probability of transmission between two individuals is proportional to the Euclidean distance between them. To divide the population into a set of *k* distinct LTZs we make use of an optimization technique known as k-means clustering that calculates the *k* different geographic zones while attempting to ensure that Eq. 1 holds in an optimal fashion^25^.

For any preassigned *k* we are able to divide the set of all patients home-locations into a set of *k* LTZs using the aforementioned clustering method, after determining all pair-wise Euclidean distance between address locations of the full patient set (see also methods). Thus the resulting LTZs represent *k* groups of patients, partitioned purely on the basis of the physical proximity between these patients’ home locations^27^,^28^. Note that when examined over the entire period (2009–12), the spatial distributions of Influenza and RSV are very similar and we have found that the LTZs obtained using only the Influenza or RSV data for clustering purposes are very similar to those obtained when using the entire data (not shown).

**Figure 1:**
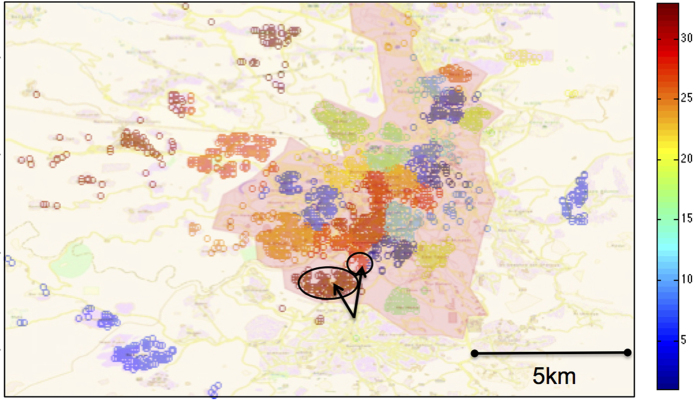
Geographic context of LTZs. The approximate home-location of all patients in the dataset (circles), shown in geographical context after dividing the set into *k* = 36 LTZs, which are identified by their respective colors; *k* = 36 is used here as a representative example of results obtained for the other *k* values tested. The shaded area represents the city municipality of Jerusalem. Arrows point to the Gilo (bottom, left) and Bet-Safafa (top, right) neighbourhoods. The cartography in the image was adapted from OpenStreetMap, licensed under CC BY-SA (www.openstreetmap.org/copyright).

The clustering method used was effective at identifying geographically distinct areas as clusters, e.g. neighborhoods outside the Jerusalem municipal boundary (Fig. 1, highlighted area). Here we chose *k* = 36 LTZ groups as a representative example because it guaranteed that the smallest LTZ consisted of at least 100 individuals. Interestingly, the clustering algorithm was also able to make meaningful distinctions within the municipal boundaries, e.g. between the ‘Bet-Safafa’ and ‘Gilo’ neighbourhoods (indicated on map as circles).

### Disease-signal overlap

**Figure 2:**
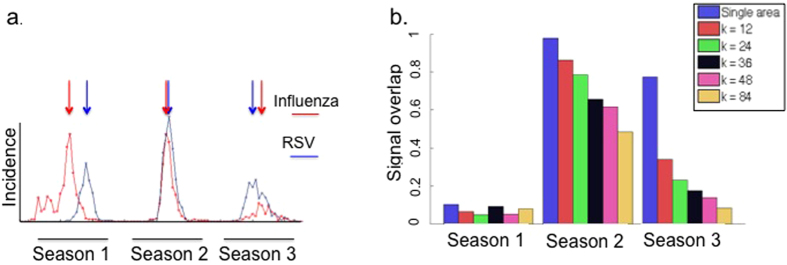
Per season signal overlap of Influenza and RSV. (**a**) Influenza (red) and RSV (blue) incidence (y-axis), summed over one-week time periods (x-axis) in the whole region. The three ‘seasons’ (2009–10, 2010–11 and 2011–12) are marked. (**b**) The signal overlap (y-axis) per season was calculated using Eq. [2] for a single area (blue) and for the different LTZ groups (*k* = 12, 24, 36, 48 and 84, indicated by color). The whole region area values were *SO* = 0.11, 0.98 and 0.78, for the first, second and third seasons respectively. The reduction in signal overlap compared to the whole region was significant for all *k* values in 2010–11 and 2011–12, but not significant during 2009–10.

The time-series of Influenza and RSV clinical test results collected at The Hadassah-Hebrew university Medical Centers between 2009 and 2012 is shown (Fig. 2a). The unusual dynamics in 2009–10 may be the result of the unusual ILI dynamics during this season, caused by the then emerging H1N1 Influenza pandemic strain (H1N1pdm, ref. 12). In the 2009–10 season, we note the occurrence of first an Influenza epidemic followed by an RSV epidemic, with very small overlap between the two epidemics. As such there is a notable time-delay between the peaks of the epidemic curves of Influenza and that of RSV. In contradistinction, over the 2010–11 season, the Influenza and RSV epidemics peak at almost the same time and overlap almost totally.

Ideally we expect to find that within a single well defined working LTZ, the two disease signals show relatively small overlap. The underlying concept is that any pathogen arriving at a susceptible LTZ is able to spread rapidly through the entire local population. This domination within an LTZ is to be expected since disease transmission within an LTZ is stronger than transmission between them. Such a situation would be particularly favoured if only a limited number of infected individuals invade a susceptible but heterogeneous region. The pathogen dominating an LTZ is likely to be the first successfully invading pathogen. In this extreme case, there would be zero overlap of the diseases in any LTZ, because each LTZ has only a single pathogen. Here, our working assumption is that transmission between LTZs has relatively minor impact at these time-scales. Hence, even if at the whole region scale (i.e. *k* = 1) disease signal overlap is high, as say in 2010–11, in the individual LTZs within the region the disease signal overlap should be expected to be far smaller, ideally close to zero.

This motivates us to develop a quantitative index for measuring disease Signal Overlap (SO). To do so, we let the number of Influenza and RSV cases at time *t* be represented by *I*(*t*) and *R*(*t*) respectively. Let *ρ*_*I*,*R*_(*τ*) be the lagged cross-correlation between *I*(*t*) and *R*(*t* − *τ*), that is, the cross-correlation between the Influenza and RSV time series when there is a time-delay *τ* between the signals. The signal overlap *SO*_*I*,*R*_ between the two time-series is then defined as (see also Methods):


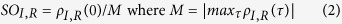


Here *ρ*_*I*,*R*_(0) is precisely the usual Pearson correlation between *I*(*t*) and *R*(*t*) and this is divided by the maximum such correlation possible when the time-series are delayed for a time *τ*, ranging from minus to plus 13 weeks.

The index is first used to examine the disease signal overlap when the whole region is considered a single LTZ (i.e., *k* = 1). The overlap for Influenza and RSV ranges from a minimal value of SO = 0.11 during the 2009–10 season, to a maximal value of SO = 0.98 in 2010–11, with the 2011–12 season also showing a high degree of overlap at SO = 0.78 (Fig. 2b). These overlap values match well what might be expected when judging by eye the overlap of the epidemic curves (Fig. 2a).

Now compare these values to the average overlap values found when the region is divided into LTZs. In the 2010–11 and 2011–12 seasons there is a remarkable decrease in the per season temporal overlap of Influenza and RSV disease signals as *k*, the number of LTZs, is increased (Fig. 2b, dark blue bars). In 2010–11 the overlap drops from SO = 0.98 (*k* = 1) to SO = 0.4 (*k* = 84) and in 2010–11 and from SO = 0.78 (*k* = 1) to less than SO = 0.1 for *k* = 84 (*p* < 0.01 for all *k* values). During the 2009–10 season, the reduction in overlap was less pronounced and not statistically significant, changing from SO = 0.11 (*k* = 1) down to SO = 0.06 (*k* = 48).

### Disease ratios

Disease signal overlap is a useful index for studying and comparing the intersection of two diseases over a season. For shorter term dynamics we make use of the disease ratio index (DR) of the weekly incidence of the two pathogens. For a particular week, the disease ratio for Influenza and RSV is defined here as (see also Methods):


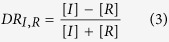


where [*I*] and [*R*] are the number of new cases of Influenza and RSV respectively, for that week.

The disease ratio quantifies the degree to which one pathogen is dominant over the other during a period of one week. When only Influenza is detected over a week, the disease ratio is *DR* = +1, when only RSV is detected then *DR* = −1, and hence the absolute value of the DR is maximal when only one pathogen is present (|*DR*| = 1). If the pathogen incidences are equal over the week then *DR* = 0.

**Figure 3:**
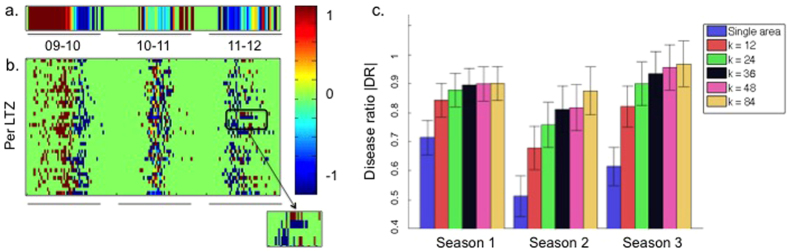
Average per week disease ratios. (**a**) Color-coded per week DR for the whole region (Jerusalem area) data; red indicates *DR* = +1, blue indicates *DR* = −1 and green indicates *DR* = 0. (**b**) Color coded per week DR for each LTZ (y-axis), with *k* = 36 as a representative example. Inset shows *DR* in 4 arbitrary LTZs during the 2011–12 season. (**c**) The average absolute |*DR*| (y-axis) per season +/− SD are shown for the whole region, *k* = 1 (blue) and the different LTZ groups (*k* = 12, 24, 36, 48 and 84, indicated by color). All changes were significant at *p* < 0.01.

We show the disease ratio for the whole region (*k* = 1) colour-coded and plotted as a function of time in consecutive weeks (Fig. 3a). In the first half of the 2009–10 season there are only cases of Influenza and *DR* = +1 (red), whereas for the second half of the season there are only RSV cases (*DR* = −1, blue). In contrast, during most of the 2010–11 season *DR* = 0 (green), as expected given the almost complete overlap of the Influenza and RSV disease signals.

In the 2011–12 season, despite the high degree of overlap in the entire season, there is initially a period of RSV dominance, reflected in a negative *DR* (blue). This dominance erodes into a disordered, mixed pattern where neither pathogen achieves strong dominance for any contiguous period.

It is informative to repeat the above qualitative examination of the whole region but in terms of its individual LTZ groups. Plotting the disease ratio for *k* = 36 LTZs as a representative example (Fig. 3b), reveals substantial variation in the *DR* of different LTZs. In the first season (2009–10), nearly all LTZs reflect the pattern observed for the *k* = 1 analysis. Namely, for the first part of the year *DR* = +1 (Influenza dominance) while for the second half of the year *DR* = −1 (RSV dominance). Note however, that the individual LTZs show a large degree of variability in terms of when the appearance of the first pathogen was detected in a patient, and when the dominance pattern changed from Influenza to RSV. The 2011–12 season shows a different phenomenon. Most importantly, even when there are roughly equal number of Influenza and RSV cases in the whole region, the DR in individual LTZs will often show a more binary pattern, with long periods where some LTZs contain only Influenza (red), while others only RSV (blue). The inset in Fig. 3b shows in detail a period of over 2 months (2011–12 season) where the single area DR for *k* = 1 is near zero while 3 of the 4 highlighted LTZs contain (almost) only RSV, and the remaining LTZ contains only Influenza consistently over the entire season.

We make these visual observations concrete by calculating the average per-season absolute disease ratio (|*DR*|) and making quantitative comparisons between analyses based on the whole area (*k* = 1) and analyses based on an LTZ approach (*k* > 1). We are particularly interested in knowing when the LTZ is dominated entirely with Influenza or dominated entirely with RSV. For either case the absolute disease ratio attains the value |*DR*| = 1.

Dividing the whole region into LTZ groups (*k* > 1) led to a significant increase (*p* < 0.01, *n* = 30, using 2-sided student t-test) in the absolute value of the disease ratios compared to the whole region (*k* = 1) results for all k values (Fig. 3c). For the 2009–10 season the ratio increased from |*DR*| = 0.7 in the whole region to |*DR*| = 0.9 using *k* > 12 LTZs. For the 2010–11 season, there was an increase from |*DR*| = 0.5 to |*DR*| = 0.8 for *k* > 24, and from |*DR*| = 0.6 to *DR* > 0.9 for *k* > 12 in the 2011–12 season. Shortly, we use the concept behind this index to assess the likelihood of someone residing in that LTZ to be infected by Influenza or infected with RSV.

### Frequency-based differentiation of Influenza and RSV

The above results show that compared to the regional level (*k* = 1), there is a reduced coexistence of Influenza and RSV when examined at the LTZ level (*k* > 1), leading to lower signal overlap (Fig. 2b), and increased disease ratios within LTZs (Fig. 3).

Together, these results support our hypothesis that patients from the same LTZ will have a strong propensity to carry the same pathogen. This suggests that the pathogen incidence within an LTZ may be better predicted by considering the data from that LTZ, rather than the more abundant, yet less specific data collected at the whole region level (*k* = 1).

We now make use of the LTZ concept to implement a test-algorithm designed to predict whether a patient has Influenza or RSV given that a person arrives at hospital with ILI symptoms, based solely on previous data kept in the hospital database. The test depends on determining the specific LTZ that the patient resides in and recent information about the pathogens present in that LTZ. If the LTZ hypothesis is correct, LTZ-based predictions should significantly outperform predictions based on the whole region data, where LTZs are ignored. If on the other hand, the LTZ hypothesis is incorrect, then an attempt to predict a patient’s disease by using LTZ-specific pathogen content would be no better than predictions where the whole region is considered as a single unit.

The test proceeds on a day-by-day basis beginning from the earliest time-point in the data (January 2009) and advances in chronological order (up to May 2012). Predictions are made on each day *t* for all patients that arrive in the hospital with ILI symptoms on that day. Predictions for a newly presenting patient arriving on day *t* are made as follows:

- The LTZ associated with the patient is determined.
- The clinical test results of all previous patients belonging to the patient’s LTZ are retrieved. The clinical test results give accurate diagnoses of all patients arriving before day *t*. This allows determination of the proportion *p* of Influenza cases in the LTZ out of the total positive test results (Influenza and RSV) over the week preceding day *t*.
- A random number *r* is drawn from a uniform distribution in the interval [0, 1]. If *r* < *p* the patient is predicted to have Influenza, otherwise the patient has RSV. For example, if *p* = 1 the algorithm will always predict the patient has Influenza.
- After making predictions for all patients over the season, the ‘Accuracy’ (*A*) of the algorithm is simply the proportion of correct predictions in the season.

**Figure 4:**
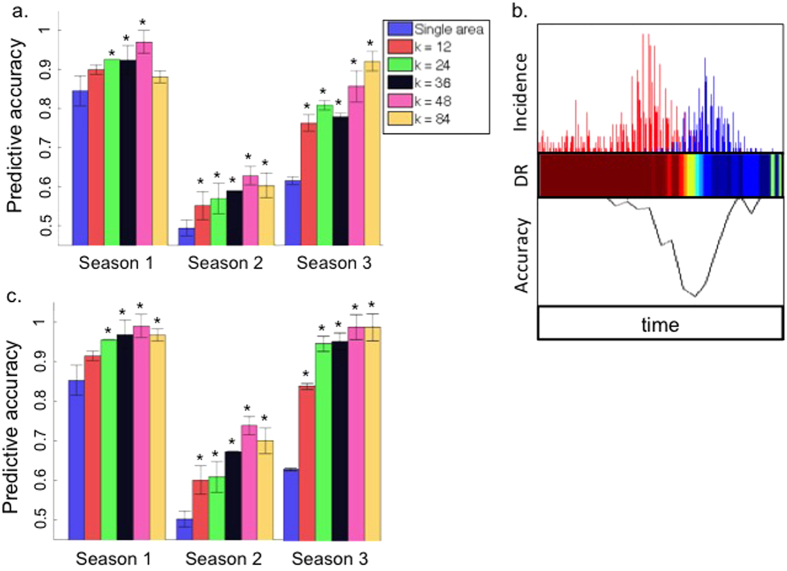
Predictive accuracy in differentiating Influenza from RSV cases. (**a**) the mean per season predictive accuracy (y-axis) for the whole region (*k* = 1) and for the LTZ groups (*k* = 12, 24, 36, 48 and 84, indicated by color). Significant improvement over the whole region results (*p* < 0.01) are marked with asterisks. (**b**) Per day incidence of Influenza(red)/RSV(blue) (top panel), per-day DR values (red = 1, blue = −1) and the per day predictive accuracy (bottom panel), during 2009–10, for k = 1. (**c**) predictive accuracy figure as in (**a**), only with the 10% worst performing LTZs removed.

It is to be expected that the per season accuracy of the predictions will improve with smaller signal overlap and increased disease ratio. The per season predictive accuracy for the three seasons as a function of the number of LTZs *k* are presented (Fig. 4a). During the first season, where signal overlap was minimal, the predictive accuracy was greater than *A* = 80% in all tests, ranging from a minimum of approximately *A* = 83% for the whole region, up to *A* = 96% for *k* = 48 LTZs. This high accuracy, even for the whole region, reflects the low signal overlap between RSV and the pandemic Influenza strain that emerged in 2009^12^.

During the 2011–12 season, the whole region (*k* = 1) predictions were accurate only in about A = 60% of cases, whereas the LTZ-based predictions (*k* > 1) reached accuracy of over A = 90%. This is consistent with what might be expected given the great reduction in signal overlap during that season for larger k values (Fig. 2b). During the 2010–11 season, where the signal overlap was almost unity, predictions based on the whole region were similar to predictions made at random, with an accuracy level of A = 50%. The LTZ-based approach (*k* > 1) preformed significantly better but only achieved an accuracy of A = 60% correct predictions (*k* = 48). The increase in predictive accuracy when using the LTZ-based approach was significant (*p* < 0.01) for all values of *k* in 2010–11 and 2011–12, but only for *k* = 24, 36 and 48 during the 2009–10 season (Fig. 4a, significant changes marked with asterisk). These results are consistent with the differences in the signal overlap between the whole region and LTZs groups, which we describe above (Fig. 2b).

In summary, the increased predictive accuracy using LTZ-data compared to the whole region data is sufficient to prove the LTZ concept, and that home-location can provide a good basis for identifying LTZs.

Increasing the value of *k* did not always improve the accuracy of the predictions; e.g. *k* = 48 performed better than *k* = 84 in 2009–10 and in 2010–11, and *k* = 24 provided better accuracy than *k* = 36 in 2011–12. However, higher |*DR*| (and lower SO) values were associated with improved predictive accuracy over a given season (compare Figs 2, 3 and 4). This observation held true on shorter time scales; in Fig. 4b we show the time-series of Influenza and RSV (red and blue, top panel), the per-day DR values (center panel, ranging from −1 to 1) and the predictive accuracies (bottom panel) during the 2009–10 season, with k = 1 as a visually clear example; the trend was similar in all 3 seasons and for all *k* values. The accuracy varied over the season, but generally periods of higher accuracy correlated with periods of a high absolute |*DR*| value, i.e. periods dominated by one of the pathogens. There was no discernible relationship between the predictive accuracy and the number of Influenza/RSV cases.

Examination of the predictive performance of individual LTZs made clear that a small proportion of LTZs were far less accurate than others; by removing 10% of the LTZs that have the worst performance, the remaining LTZs, representing approx. 90% of patients, provide predictions that are nearly 100% accurate (Fig. 4c) for the 2009–10 and 2011–12 seasons (*k* = 36, 48, 84).

While the majority (approx. 60%) of LTZs included in the top 90% (Fig. 4c) were common to all 3 seasons, we did not find any obvious characteristics that distinguish these LTZs from those included in the 10% worst performers. Poor predictive accuracy in a specific LTZ might be the result of limitations in the data itself, or difficulties in achieving perfect clustering by the k-means clustering method, i.e. it is quite possible that some LTZs were poorly identified or in fact ‘misidentified’ due to errors introduced by the k-means clustering^25^.

## Discussion

This study is the first of its kind in using a combination of clinical and geographical data to demonstrate that from a ‘pathogen’s perspective’ even a geographic unit as small as one urban area is not a single, perfectly mixed ecology, but is in fact comprised of more basic transmission components, i.e. LTZs, which should be seen as tightly connected micro-community structures that allow for rapid pathogen transmission.

Specifically, we proposed the hypothesis that in terms of the spread of ILI, the city of Jerusalem and its surrounding areas are not a single disease transmission unit but may be more appropriately described by a set of multiple smaller-scale component LTZs. These LTZs, due to spatial fragmentation and isolation effects, will tend to have extended time-periods in which only a single disease dominates, often completely.

To test this hypothesis we examined the geographic distribution of Influenza and RSV cases in Jerusalem over 3 seasons, from 2009–10 to 2011–12. Analysis of the putative LTZ groups showed unusually small Signal Overlap and large absolute Disease Ratio (Figs 2 and 3) as compared to the whole region values (i.e. *k* = 1). These results show that the spatial distribution of Influenza and RSV incidences may be better differentiated in location-based groups of patients than it is in the entire area as a whole. This confirms our hypothesis that the Jerusalem region is comprised of multiple LTZs, and is not by itself a single transmission unit.

We also measured the performance of a simple algorithm for predicting whether incoming hospital patients with ILI symptoms were infected with RSV or Influenza. The prediction scheme made use of a frequency based approach and at the regional (*k* = 1) level, the predictions simply matched the relative prevalence of the diseases. Dividing the whole region into LTZs led to a substantial increase in predictive accuracy in all seasons (Fig. 4a) and moreover, after taking into account that a proportion (10%) of the LTZs used may have been misidentified, when removed the predictions for two of the seasons attained routine prediction accuracy often of 100% in differentiating between cases identified as associated with Influenza and those that were associated with RSV (Fig. 4c).

An important part of the work preformed by hospitals and health organization consists of the diagnosis and surveillance of communicable disease, which may be carried out at the molecular and/or the symptomatic levels. The results presented here hint at the potential epidemiological importance in examining disease data at the LTZ scale,and the possibility for generating ‘maps’ of the real-time distribution of pathogens at resolution levels sufficient to guide improved public health policies. Furthermore, the improved predictive accuracy achieved by focusing the analysis at the patient-specific LTZs (Fig. 4) indicates a potential utility in data-driven diagnostics, where more sophisticated algorithms taking into account LTZ information as well as other relevant population parameters, such as the patients’ age, medical background etc. is expected to further increase the predictive accuracy. We hope to explore this further in future research.

Our choice of LTZs was based entirely on patients’ approximate home-locations, without any reference to urban motifs, e.g. socioeconomic divisions, public transportation etc.^28^,^29^ and clearly provides only a partial picture of the complexity of urban transmission ecology. The social and spatial structures differ among cities, and Jerusalem may not be representative of any ‘generic’ city; e.g. it might be that in some areas the population connectivity pattern is far more homogenous, and hence the ability to leverage LTZ-based identification of a pathogen might be limited, or provide better results at other values of *k*. The “optimal value” of *k* will be the true number of LTZs in a given area, and for a given choice of pathogens, and as such is expected to vary. Unfortunately, there is no simple way of determining this optimal *k* in advance of the analysis, which is a key problem with many clustering algorithms and “community detection” network algorithms in particular^26^.

It would be of great interest to test the methodology presented here in other geographical locations, on a more comprehensive list of pathogens, and include a wider dataset of human social activities^20^,^27^,^28^,^30^, which together could provide a finer, and more dynamic definition of area- and pathogen-specific real life LTZs.

## Methods

### Settings

The study was performed using data obtained from the computerized database of Hadassah-Hebrew University Medical Center, Jerusalem, Israel, a tertiary care medical institution serving a population of approximately 1 million people from the entire Jerusalem metropolitan. The demographics and microbiological data included in this study were collected from the institutional database between January 2009 and May 2012. The dataset contained 16,000 positive and negative clinical test results for two causing agents: Influenza virus (Influenza) and RSV. The area considered in the analysis is a square shape of approximately 700 sq. km. with vertices at: (32, 35); (31.6, 35); (31.6, 35.4); (32, 35.4). The whole Jerusalem urban area population is well covered by this sample since the hospital provides care to over 60% of the referrals from the population in all parts of the city homogeneously.

Diagnosis of both Influenza and RSV at the Hadassah-Hebrew university was done on routine basis for patients arriving either to the emergency room or being admitted. The diagnosis was based on direct immunofluorescence assay using commercial monoclonal antibodies (Chemicon, Temecula, California) until 7th March 2010, and by in house RT-PCR assay after that date^31^.

The original data contained patient addresses in free text format collected from patients during admission (e.g. ‘house number **x**, street **y**, town **z**’), which were converted into standard coordinate system using a process of reverse geocoding. In order to maintain patient anonymity the last 3 digits of the obtained coordinates were omitted, resulting in a location resolution of 110 on 110 meters. The study was approved by the Hadassah institutional ethics committee.

### Clustering

Patients were partitioned into *k* groups using the k-means clustering method^25^, applied to the (approximate) home locations of the entire patient set.

The minimal number of individuals in any LTZ was reduced as *k* increased, however at the highest k value tested (*k* = 84) there was still a minimum number of 37 individuals in any LTZ (with minimum numbers of 776, 196, 121, and 55 individuals for *k* = 12, 24, 36, and 48, respectively). Thus our schemes provides reasonably sufficient data for statistical estimates even for k-values as large as *k* = 84.

The *k* values tested were arbitrarily set to multiples of twelve. Results for the values *k* = 60 and *k* = 72, or of other intermediate k-values we tested (e.g., *k* = 13 or *k* = 31) provided qualitatively similar results and are not presented in this study for clarity. Note the number of meaningful clusters that may be found is limited by data type/availability. As *k* is increased, more data is required to accurately extract LTZs. This is because a minimum number of data-points is needed in an LTZ before it can be identified accurately by the algorithm.

Adding a temporal element to the clustering process would be critical for detecting “pathogen hotspots” (using SatScan, www.satscan.org, Kulldorff, Harvard Medical School, Boston, MA), typically characterized by a significantly elevated incidence of one disease/pathogen. There is no question that detection of disease hotspots is an important activity in modern disease surveillance where it may be used as a real-time warning alert of any atypical activity. Our aims however are quite different, since here our goal is to determine if a city-area (i.e. Jerusalem) is essentially a single, well-mixed ecology in terms of pathogen transmission, or if there are additional spatial partitions within this limited geographic scope. To address the question in the most direct way, we make use of the robust k-means clustering algorithm, based only on proximity between home locations addresses.

### Disease-signal overlap

We define the general form of a disease signal (DS) for some causing agent *v* as a function:


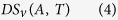


*DS*_*v*_(*A, T*) is the number of positive cases of infection *v* in a monitored area *A* over a time period *T*. For our analysis below, *v* is a boolean variable, where 1 denotes the Influenza virus and 2 denotes RSV. From the data available to us, this function can be immediately constructed for any chosen area *A* and time period *T*. The time period *T* is usually defined to be a week at a time, hence *DS*_*v*_(*A, T*) represents the total number of positive cases of disease *v* in area *A* during week *T*. We choose 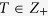. Our data runs from January 2009 to May 2012, and hence 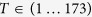.

Generically, any area analyzed may contain different disease signals at (or over) the same (period of) time, e.g. 10 positive clinical test results for Influenza and 10 for RSV. In this case the signals from the *v* = 1, 2 are termed ‘overlapping’. We calculate the per season overlap *SO*_*v*1,*v*2_ of two disease signals *v*1 and *v*2 using the following methodology:


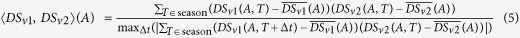


Here 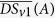 is the average over the season of *DS*_*v*1_(*A, T*), and where the maximization is done over a time-shift 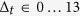 weeks, spanning all events over each entire season. This is also called the ratio of the cross correlation coefficients of the unnormalized vectors *DS*_*v*1_(*A, T*) and *DS*_*v*2_(*A, T*): 
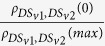
, which is a quantity depending on the area in question (see Eq. 2, main text). The maximization over the time-shift Δ*t* is done in absolute value, i.e. we are maximizing the absolute value of the time-shifted cross correlation. In the context of the current paper *v*1 and *v*2 represent Influenza and RSV respectively.

The resulting degree of signal overlap per a given period varies between −1 and 1, where 1 indicates *DS*_*v*1_ and *DS*_*v*2_ completely overlap (same frequency) and zero time shift, and a −1 score indicates anticorrelation and zero time shift. The overlap is calculated per period, in the results presented here, per ‘winter season’, defined here as August year *X* to May year *X* + 1, that is three seasons in total: 2009–10, 2010–11, and 2011–12. Notice that our definition of the ratio in Eq. [5] is different from the usual cross correlation: a score of 1 implies two possibilities: 1. that the data is perfectly correlated at zero time shift or 2. that the data is perfectly correlated and periodic with some period Δ*t*.

By dividing the whole region into *k* 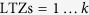 of area 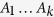, we can compute the signal in Eq. [5] for each LTZ. This provides a more local way to analyze the correlations between diseases. The total signal is then the weighted sum of the signal of the individual LTZs with weight factors *W*_*i*_ equal to the percentage population size *N*_*i*_ of the *i*’ th LTZ:


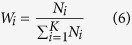


We define the resulting overall signal overlap as a weighted sum over all LTZs that comprise the area analyzed.

Given some (number *n* of) LTZs 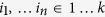, of areas 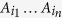, we define a resulting total signal overlap in area 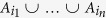 as a weighted sum over the LTZs considered:


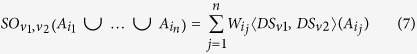


The signal overlap in each LTZ, and consequently the overall ratio in the whole region are bound between negative one - anticorrelation) and one (complete overlap).

### Disease signal ratios

We define the relative disease ratio *DR* of the incidence of two viruses using the following formula:


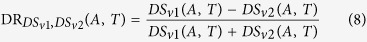


The relative signal ratios are bound between a value of one (all cases are Influenza) and negative one (all cases are RSV) and are specific per time-period, here calculated per one week time periods; when the incidence of Influenza is equal to that of RSV the ratio is equal to zero.

The per-season absolute |*DR*| values (presented in Fig. 3c) were calculated as the average of the absolute values of the per-week |*DR*| values; when the per-week incidences of Influenza and RSV are identical, the average value is minimal, i.e. zero, and when Influenza and RSV do not coincide on any week the average |*DR*| is 1. When calculating the per week relative disease ratio over multiple LTZs, the overall ratio is the weighted average of the ratios in the LTZs.

## Additional Information

**How to cite this article**: Almogy, G. *et al*. Analysis of Influenza and RSV dynamics in the community using a ‘Local Transmission Zone’ approach. *Sci. Rep.* **7**, 42012; doi: 10.1038/srep42012 (2017).

**Publisher's note:** Springer Nature remains neutral with regard to jurisdictional claims in published maps and institutional affiliations.
